# Compound Congrong Yizhi capsule partially remodels the fronto-retrosplenial-hippocampal memory circuit in vascular dementia rats

**DOI:** 10.3389/fneur.2026.1867157

**Published:** 2026-09-01

**Authors:** Ying Zhang, Yefei Wang, Xuemei Liu, Baoxin Chen, Shaozhen Ji, Jiayu Duan, Junya Liao, Yihan Wang, Xianglan Jin

**Affiliations:** 1 Graduate School, Beijing University of Chinese Medicine, Beijing, China; 2 Department of Neurology, Dongfang Hospital, Beijing University of Chinese Medicine, Beijing, China

**Keywords:** Compound Congrong Yizhi capsule, disconnection syndrome, fronto-retrosplenial-hippocampal memory circuit, resting-state functional magnetic resonance imaging, vascular dementia

## Abstract

**Background:**

Progressive cognitive impairment in vascular dementia (VaD) is increasingly linked to brain network disruption, or “disconnection syndrome.” Although Compound Congrong Yizhi Capsule (CCYC), a marketed Chinese patent medicine approved in China for vascular cognitive disorders, has been studied in terms of therapeutic efficacy and molecular targets, these approaches do not directly explain how this multi-target intervention modulates brain functional organization. Resting-state functional magnetic resonance imaging (rs-fMRI) enables *in vivo* characterization of BOLD-based brain network functional phenotypes. Here, we used rs-fMRI to investigate the effects of CCYC in a rat model of VaD.

**Methods:**

VaD was induced by permanent bilateral common carotid artery occlusion (BCCAO) in rats. CCYC was administered by gavage for 6 weeks. Spatial navigation and memory retention were assessed using the Morris water maze (MWM). Resting-state fMRI was acquired on a 9.4 T scanner and analyzed using regional activity metrics, behavioral correlations, functional connectivity, and graph theoretical analyses.

**Results:**

VaD rats showed abnormal regional activity in the frontal cortex, retrosplenial cortex, and hippocampal system, which were associated with impaired MWM performance. They also exhibited disrupted connectivity within the fronto-retrosplenial-hippocampal memory circuit, particularly between prefrontal/cingulate and retrosplenial-hippocampal regions. CCYC treatment was associated with partial normalization of these regional and circuit-level abnormalities. Graph theoretical analyses further showed partial improvement in whole-brain topological organization and nodal properties after CCYC treatment. These findings do not establish anatomical restoration of the underlying white matter tracts.

**Conclusion:**

CCYC may ameliorate VaD by modulating a systems-level neuroimaging phenotype centered on the fronto-retrosplenial-hippocampal memory circuit.

## Graphical abstract

**Figure:**
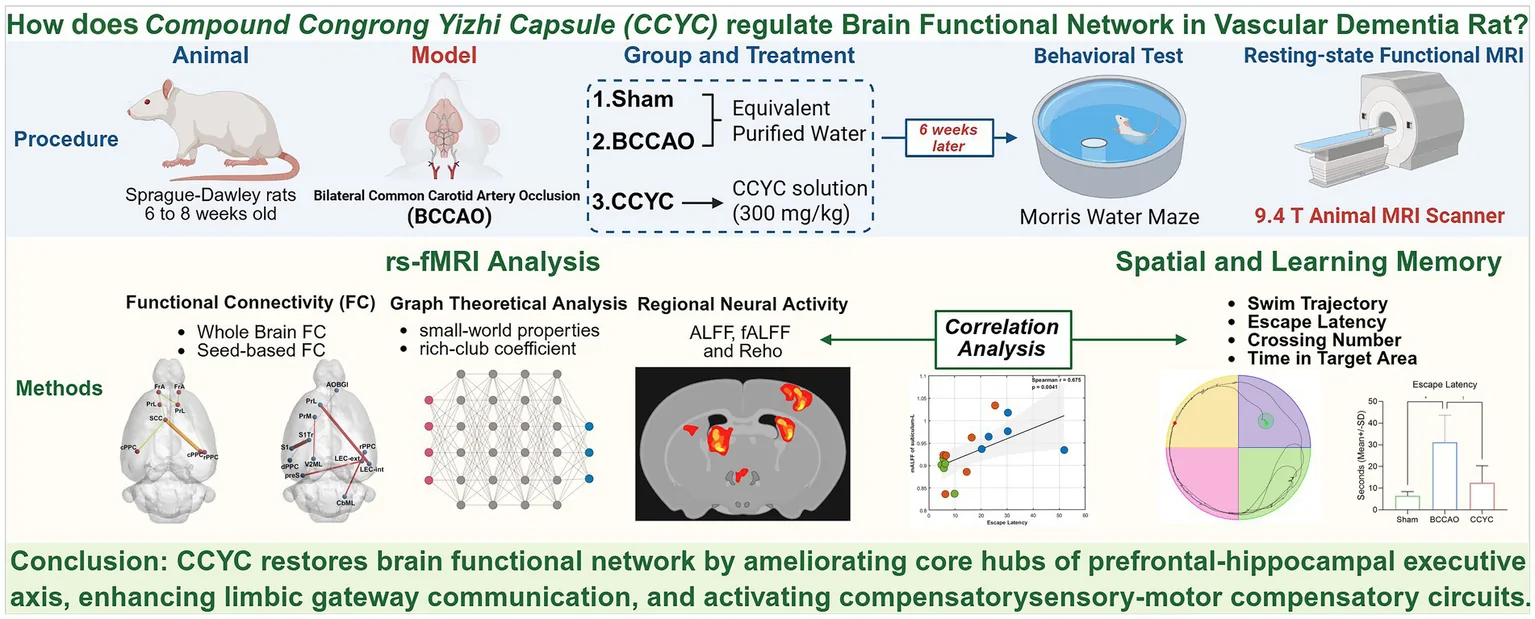
Created in BioRender. Wang, Y. (2026) https://BioRender.com/ulgfedd.

## 1 Introduction

Vascular Dementia (VaD) is fundamentally characterized by the gradual loss of cognitive integrity and memory, following chronic insufficiency of the cerebral blood supply (1). VaD is the second most common type of dementia after Alzheimer’s disease (AD), accounting for approximately 15–20% of all dementia cases, with an even higher incidence in Asian regions (1, 2). Increasing evidence suggests that cognitive impairment in VaD is associated with disruption of large-scale functional brain networks, resulting in impaired communication and information integration among distributed cognitive regions (3, 4). From a functional-network perspective, this pattern has been discussed within the broader framework of a “disconnection syndrome,” reflecting impaired coordination among memory- and executive-related regions (4–7). Chronic cerebral hypoperfusion (CCH) may contribute to this network-level dysfunction by altering regional brain activity, interregional coupling, and information-transfer efficiency. In rodent studies, bilateral common carotid artery occlusion (BCCAO) is widely used to model CCH-related cognitive impairment and reproduces deficits in spatial learning, memory, and executive function (8).

Resting-state functional Magnetic Resonance Imaging (rs-fMRI) provides a non-invasive approach for characterizing this systems-level neuroimaging phenotype *in vivo*. By analyzing the spontaneous synchronization of blood-oxygenation-level-dependent (BOLD) signals in different brain regions, such as amplitude of low-frequency fluctuations (ALFF) and regional homogeneity (ReHo), we can assess the intrinsic functional organization of the brain, like spontaneous neuronal activity and local synchronization (9, 10). Functional connectivity (FC) captures the statistical relationships within and between neuronal networks of brain regions (11). Graph theory provides a quantitative framework for evaluating nodal-information of transfer efficiency by conceptualizing the neural architecture as a set of nodes and edges (12). Together, these approaches make it possible to examine VaD-related functional abnormalities from regional, circuit, and global network perspectives.

Current therapeutic options for VaD remain limited. Compound Congrong Yizhi Capsule (CCYC; originally developed as Congsheng Capsule) is a marketed Chinese patent medicine approved in China for VaD (Guoyao Zhunzi No. Z20194044), and its clinical use has also been supported by Chinese expert consensus (No. GS/CACM 298-2022) (13). Previous clinical studies suggested that CCYC might improve cognitive function and daily living ability in patients with VaD (14, 15). Preliminary research from our group found that after 6 weeks of treatment, compared with the BCCAO, the CCYC group attenuated chronic hypoxia-related microvascular injury and modulated angiogenesis- and hypoxia-related pathways, including hypoxia-inducible factor-1α (HIF-1α) and Vascular Endothelial Growth Factor (VEGF) (16). However, despite these encouraging clinical and mechanistic findings, previous studies on CCYC have largely focused on therapeutic efficacy and target-level mechanisms, which are insufficient to explain how the intervention reshapes brain regional activity, circuit interactions, and global network organization in VaD. Because VaD is increasingly recognized as a network-based disorder, evaluation at the level of systems neuroimaging phenotypes is therefore necessary.

In the present study, we used BOLD rs-fMRI to examine functional brain network alterations in BCCAO rats and to assess the effects of CCYC intervention, while also exploring the relationships between imaging metrics and behavioral performance. Through this approach, we aimed to delineate the modulation of functional network in VaD and to further clarify the network-level mechanisms underlying the effects of CCYC from a multi-scale perspective.

## 2 Methods

### 2.1 Animals and experimental procedure

A total of 16 male Sprague–Dawley (SD) rats (6–8 weeks, 180–220 g) were acclimatized for 1 week and assigned using a random-number table to three groups: sham-operated group (SHAM, *n* = 5), bilateral common carotid artery occlusion group (BCCAO, *n* = 5), and CCYC-treated group (CCYC, *n* = 6). Investigators performing the Morris water maze tests and brain MRI analysis were blinded to the group allocations. No animals or imaging datasets were excluded from the study and final analysis. Sample size was determined with reference to the HIF-1*α* results from our preliminary experiments (*α* = 0.05, power = 0.80, G * Power) (16), and previous small-animal BOLD-fMRI studies with similar designs (17).

All rats were maintained at the specific-pathogen-free (SPF) grade animal facility (Dongfang Hospital, Beijing University of Chinese Medicine), under a 12-h light/dark cycle (lights on at 06:00). The housing environment remained stable at 23 ± 2 °C and 50% ± 10% humidity. All procedures were approved by the Institutional Animal Care and Use Committee of Dongfang Hospital, Beijing University of Chinese Medicine (Approval No. DFYY202303R).

After a one-week adaptive period, artery occlusion was performed in the BCCAO and CCYC group. Isoflurane was used for inhalation anesthesia (4% for induction, 1.5% for maintenance). Following a midline cervical incision, both common carotid arteries were exposed and carefully separated from the vagus nerves via blunt dissection. To induce chronic ischemia, we permanently ligated the arteries using 1–0 silk sutures (8). Post-surgery, animals were monitored daily for health status and weight changes. Meloxicam (1 mg/kg s.c.) was administered for post-operative analgesia to minimize suffering. Rats in the SHAM group was subjected to the same operative environment and tissue manipulation, barring the actual arterial ligation.

Commencing 24 h after the operation, rats in the CCYC group received an oral gavage of 300 mg/kg/day (CCYC Batch No. 241222). The selection of this monotherapy dose had been justified by preliminary dose-finding studies (16).

This optimized dose was utilized for all subsequent longitudinal investigations. The capsules were dissolved in purified water, and delivered via gavage (0.1 mL/100 g). Purified water was given to the SHAM and model rats at an identical volume. Oral administration was conducted on a daily basis for 6 weeks in succession.

### 2.2 Compositions of CCYC

CCYC is clinically administered in capsule form. It is composed of five Chinese medicines: *Fallopia multiflora* (Thunb.) Haraldson (33.32%), *Nelumbo nucifera* Gaertn. (16.68%), *Cistanche deserticola* Y.C.Ma (10%), Dilong *Pheretimaas pergillum* (E Perrier) (23.32%), and *Rhaponticum uniflorum* (L.) DC (16.68%) (13). These capsules containing all the natural products above were processed via advanced micronization technology, strictly adhering to the quality control protocols and marker compound standards of the People*’*s Republic of China national drug Standard YBZ00852008. For experimental administration, the capsule contents were extracted and dissolved in purified water to prepare the working solution.

### 2.3 Chemical characterization of CCYC

**Figure 1:**
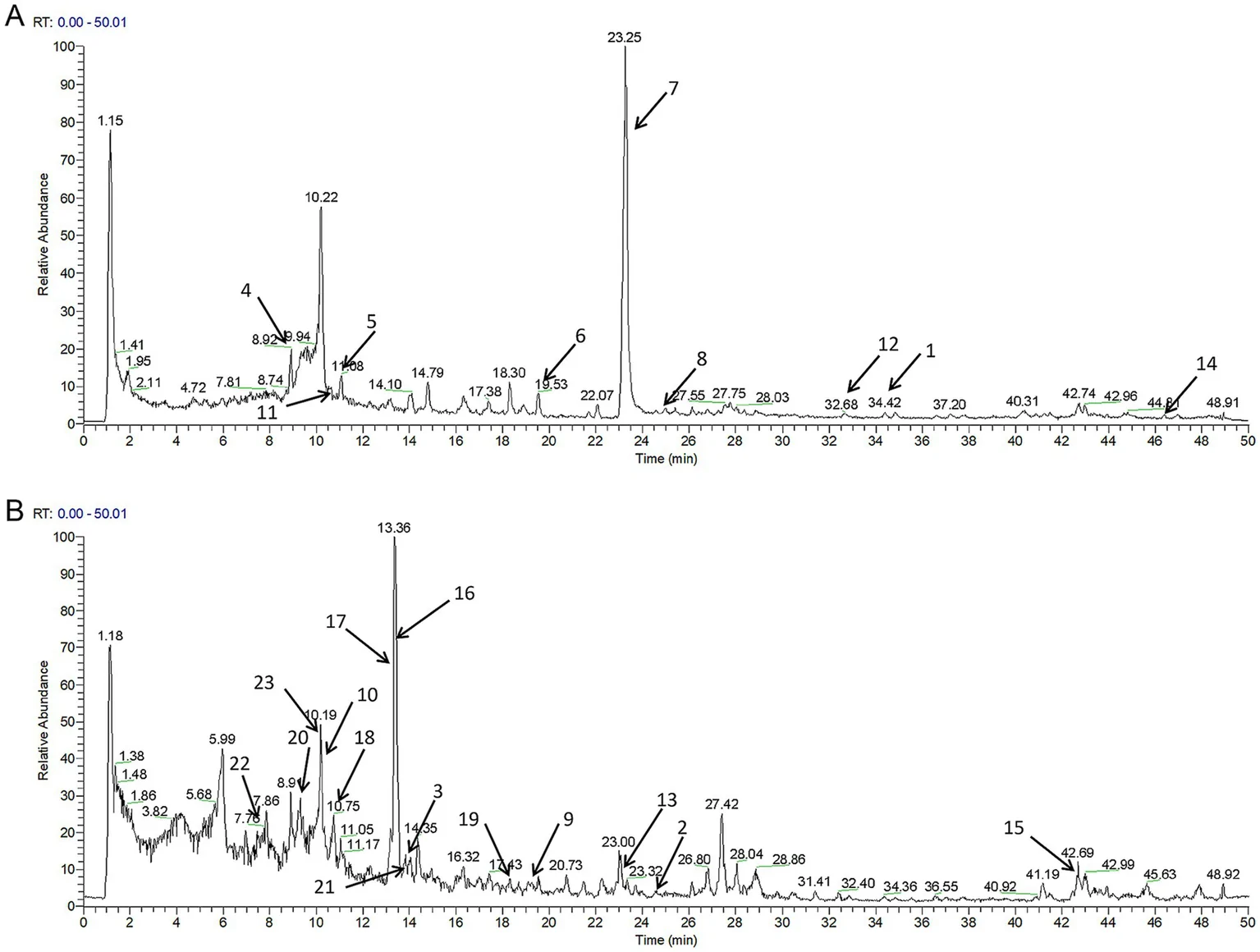
UPLC-Q-Exactive-MS/MS total ion chromatograms (TIC) of CCYC. The chemical profiling of CCYC was performed in both positive **(A)** and negative **(B)** ESI modes. A total of 23 annotated compounds were identified, including: Arachidonate (peak 1), Suchilactone (peak 2), Quercetin (peak 3), Echinacoside (peak 4), Acteoside (peak 5), Physcion (peak 6), Emodin (peak 7), Citreorosein (peak 8), Questinol (peak 9), Liquiritigenin (peak 10), Liquiritin (peak 11), Eicosapentaenoic acid (peak 12), Linolenic acid (peak 13), Myristic acid (peak 14), Cycloartenol (peak 15), Remerin (peak 16), Nuciferin (peak 17), O-Nornuciferine (peak 18), Isorhamnetin (peak 19), Armepavine (peak 20), Kaempferol (peak 21), Machiline (peak 22), (1S)-1-(4-hydroxybenzyl)-2-methyl-3,4-dihydro-1H-isoquinoline-6,7-diol (peak 23), respectively. RT, Retention Times.

CCYC formulation was provided by Lei Yun Shang Pharmaceutical Group Co., Ltd. For mobile phase preparation, analytical reagents including acetonitrile (LC/MS-grade) and methanol (HPLC-grade) were procured from Merck (Darmstadt, Germany), while formic acid (CNW) and distilled water (Watsons) were utilized as received. Chemical profiling was performed using UPLC–Q Exactive MS/MS. Chromatographic separation was achieved on a Thermo Hypersil Gold C18 column (3 μm, 2.1 × 100 mm) using water containing 2 mmol/L ammonium formate and 0.1% formic acid and acetonitrile as the mobile phases. Mass spectrometry (MS) was conducted in both positive and negative electrospray ionization (ESI) modes. Based on retention time, exact mass, and MS/MS fragmentation patterns, 23 constituents were tentatively identified. Detailed results are shown in Figure 1 and Supplementary Table S1.

### 2.4 Morris water maze (MWM) experiment

The MWM system was used to assess spatial navigation and memory retention (Beijing Zhongshi Dicuang Science and Technology Development Co., Ltd.). The apparatus featured a pool with four quadrants, containing a platform (11 cm height) submerged 1 cm beneath the water surface. One day before formal testing, rats were permitted to swim for 2 min without the platform. Training commenced in the 6th week post-treatment. For 5 days, rats underwent two times of trials (with an interval of at least 15 min). In each trial, rats were allowed 120 s to find the hidden platform submerged 1 cm below the water surface. Rats that failed to locate the platform were guided to it and assigned an escape latency of 120 s. After the navigation test, the platform was removed for the probe trial. Escape latency, crossing number, and time in target quadrant were recorded using an automated video-tracking system.

### 2.5 Image acquisition

MRI scans were performed at the Institute of Automation, Chinese Academy of Sciences. Anesthesia was induced with 4% isoflurane and maintained with 1.0–1.5% isoflurane in medical air. Rats were positioned in a heated MRI-compatible stereotaxic frame with ear and tooth bars, and respiration was monitored throughout scanning.

All images were acquired on a 9.4 T animal MRI scanner (Bruker Biospin GmbH, Germany) using a volume transmit coil and a surface receive coil. High-resolution T2-weighted anatomical images were acquired with a turboRARE sequence, followed by resting-state BOLD-fMRI using a multi-slice gradient-echo EPI sequence. Functional images were acquired with TR = 3,000 ms, TE = 6.838 ms, flip angle = 30°, 140 volumes, and an isotropic voxel size of 0.30 mm. Detailed MRI acquisition parameters are provided in the Supplementary material S2.

### 2.6 Image preprocessing

Raw DICOM images were transformed into the NIfTI format via the dcm2niix tool^1^ (18). The rs-fMRI data underwent preprocessing steps within the SPM12^2^ (19). The first five volumes were discarded, followed by slice-timing correction, realignment, co-registration, normalization to the SIGMA EPI template aligned with the Paxinos and Watson rat brain atlas, resampling to 0.3 × 0.3 × 0.3 mm^3^, spatial smoothing (FWHM = 0.6 mm), linear detrending, and band-pass filtering (0.01–0.1 Hz) (20). Detailed preprocessing procedures are provided in the Supplementary material S3.

### 2.7 Rs-fMRI analyses

Several rs-fMRI metrics, including ALFF, fALFF and ReHo, were extracted using the DPABI v9.0^3^ (21). The Resliced SIGMA Anatomical Brain Atlas was used to parcellate the brain into 158 ROIs (20). These metrics were first computed on a voxel-wise basis throughout the whole brain. Mean regional values of ALFF, fALFF, and ReHo were extracted for each ROI. Group differences in mALFF, mfALFF, and mReHo were assessed using two-sample *t*-tests in SPSS 23.0, with False Discovery Rate (*FDR*) correction for multiple comparisons across ROIs (*P_FDR* < 0.05). Detailed calculation procedures are provided in the Supplementary material S4.

### 2.8 Functional connectivity

Whole-brain ROI-to-ROI functional connectivity was calculated using the 158 ROIs of the SIGMA atlas. Mean BOLD time series were extracted for each ROI and Pearson correlation coefficients between all ROI pairs were transformed to Fisher’s *z* values. Group differences in whole-brain connectivity were assessed using Network-Based Statistic (*NBS*) v1.2^4^ (22). Seed-based functional connectivity analyses were also performed for selected ROIs. Group differences were assessed using two-sample *t*-tests with *FDR* correction (*P_FDR* < 0.05). Because some comparisons did not survive correction, exploratory results at uncorrected *p* < 0.001 are reported separately. Detailed FC procedures are provided in the Supplementary material S5.

### 2.9 Graph theoretical analyses

To evaluate the brain network topological properties, we performed graph theoretical analyses via the GRETNA 2.0 toolbox^5^ (23). The 158 ROIs were defined as nodes, and Fisher*’*s z-transformed ROI-to-ROI correlations were used to construct functional connectivity matrices. Network properties were evaluated across a range of *sparsity* thresholds (0.05–0.40, step = 0.02) in both weighted and binary networks (24).

Nodal metrics included centrality, clustering, efficiency, and path length-related measures. Nodal metrics were summarized using the area under the curve (AUC), and group differences were assessed using two-sample *t*-tests. Results that did not survive *FDR* correction are presented as exploratory findings with accompanying effect sizes. We calculated the effect size (Cohen’s *d*) to identify robust trends power. Detailed graph-theoretical procedures are provided in Supplementary material S6.

### 2.10 Correlation analyses between MWM performance and rs-MRI metrics

Across 16 rats with paired MWM and MRI data, correlation analyses were conducted to determine how regional neural dynamics relate to spatial memory capabilities. We performed Spearman correlation analyses linking the fMRI metrics (mALFF, mfALFF, and mReHo) of 158 ROIs to behavioral indices derived from the MWM (escape latency, crossing number, and time in target quadrant). Multiple comparison correction was achieved via the FDR correction (*P_FDR* < 0.05). The strength of the associations was categorized based on the calculated *r*-values, strong correlations were defined by coefficients ranging from 0.75 to 1.00 (or −0.75 to −1.00).

## 3 Results

### 3.1 Chemical profiling of CCYC by UPLC-Q-Exactive-MS/MS

UPLC-Q-Exactive-MS/MS analysis of CCYC performed in both positive and negative ion modes identified a total of 23 chemical constituents. These constituents mainly comprised phenylethanoid glycosides, flavonoids, anthraquinones, alkaloids, fatty acids, and triterpenoid-like compounds. Representative compounds included echinacoside, acteoside, quercetin, kaempferol, emodin, physcion, nuciferine, and eicosapentaenoic acid. See Supplementary Table S1, Figure 1.

### 3.2 CCYC improves learning and memory ability of VaD rats

**Figure 2:**
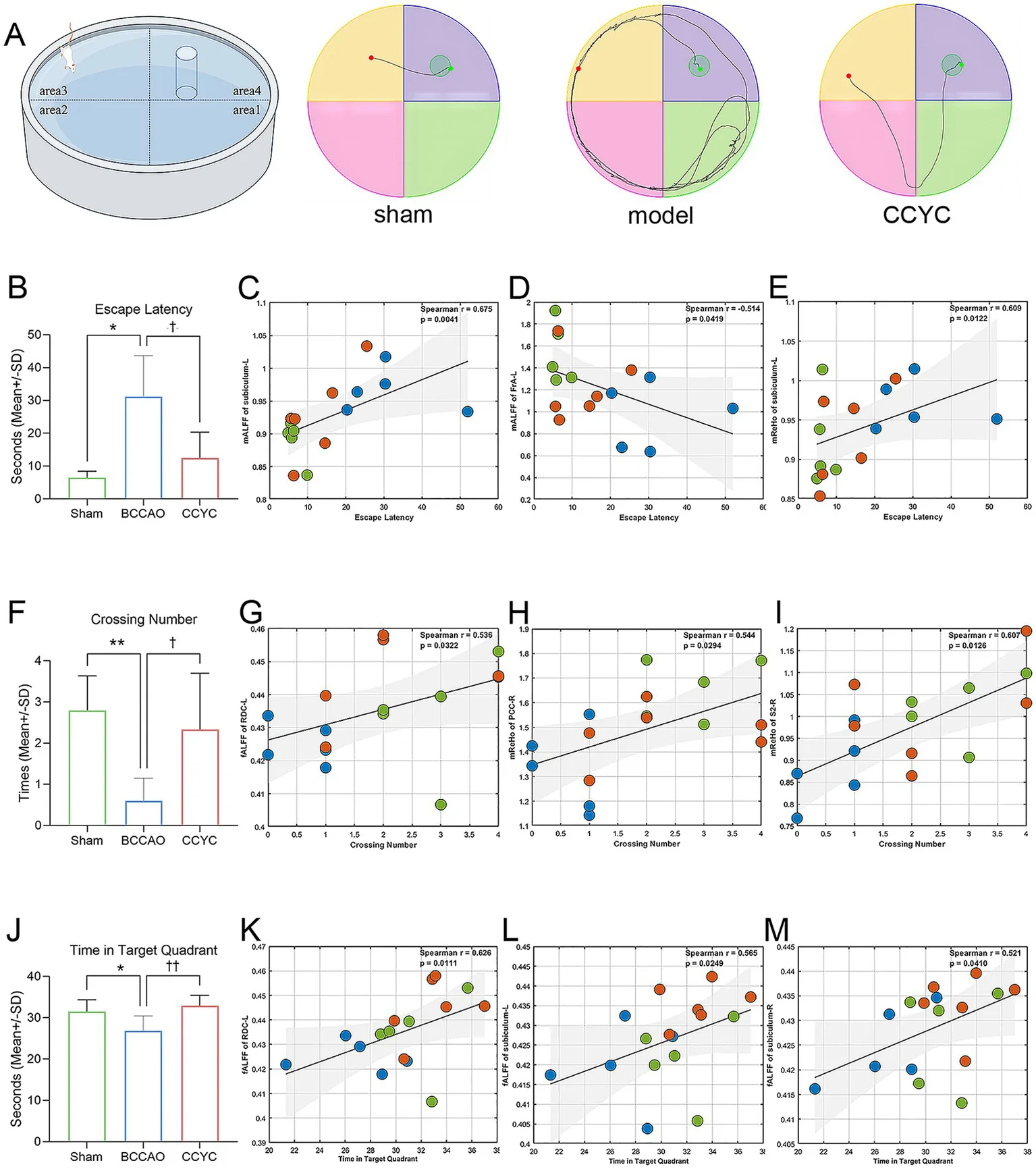
CCYC treatment ameliorates spatial learning and memory deficits in VaD rats by modulating regional spontaneous neural activity, as evidenced by significant correlations between MWM performance and localized ALFF/ReHo metrics. **(A)** The schematic diagram shows the platform of MWM is in area 4 and the rat is placed into the water from area 2. The following 3 pictures exhibit the swimming trajectories of the SHAM, BCCAO and CCYC groups. **(B, F, J)** Escape latency, crossing number and time in target quadrant are represented by the corresponding bar charts. Compare to the SHAM group, *p < 0.05, **p < 0.01. Compare to the BCCAO group, †*p* < 0.05, ††*p* < 0.01. **(C, D, E)** Correlation analyses between escape latency and spontaneous neural activity metrics. **(G, H, I)** Correlation analyses between crossing number and spontaneous neural activity metrics. **(K, L, M)** Correlation analyses between time in target quadrant and spontaneous neural activity metrics. Multiple comparison correction was achieved via the FDR correction (*P_FDR* < 0.05). In the scatter plot green spots represent the SHAM group, blue spots represent the BCCAO group and red spots represent the CCYC group. FrA, Frontal Association Cortex; PCC, Primary Cingular Cortex; RDC, Retrosplenial Dysgranular Cortex; S2, Secondary Somatosensory Cortex.

To validate the establishment of the BCCAO model and to assess the therapeutic efficacy of CCYC on cognitive function, we performed the MWM test. Compared with the SHAM group, escape latency of the BCCAO group was significantly prolonged (Cohen’s *d* = −1.5890, *p* = 0.0241), crossing number was significantly decreased (Cohen’s *d* = 3.1113, *p* = 0.008), time in target quadrant was significantly decreased (Cohen’s *d* = 1.4637, *p* = 0.040). Compared with the BCCAO group, escape latency of the CCYC group was significantly shortened (Cohen’s *d* = 1.3463, *p* = 0.0490), crossing number was significantly increased (Cohen’s *d* = −1.6022, *p* = 0.0317), time in target quadrant was significantly increased (Cohen’s *d* = −1.9803, *p* = 0.0096). See Figures 2A,B,F,J.

### 3.3 Impact of CCYC on regional neural activity

**Figure 3:**
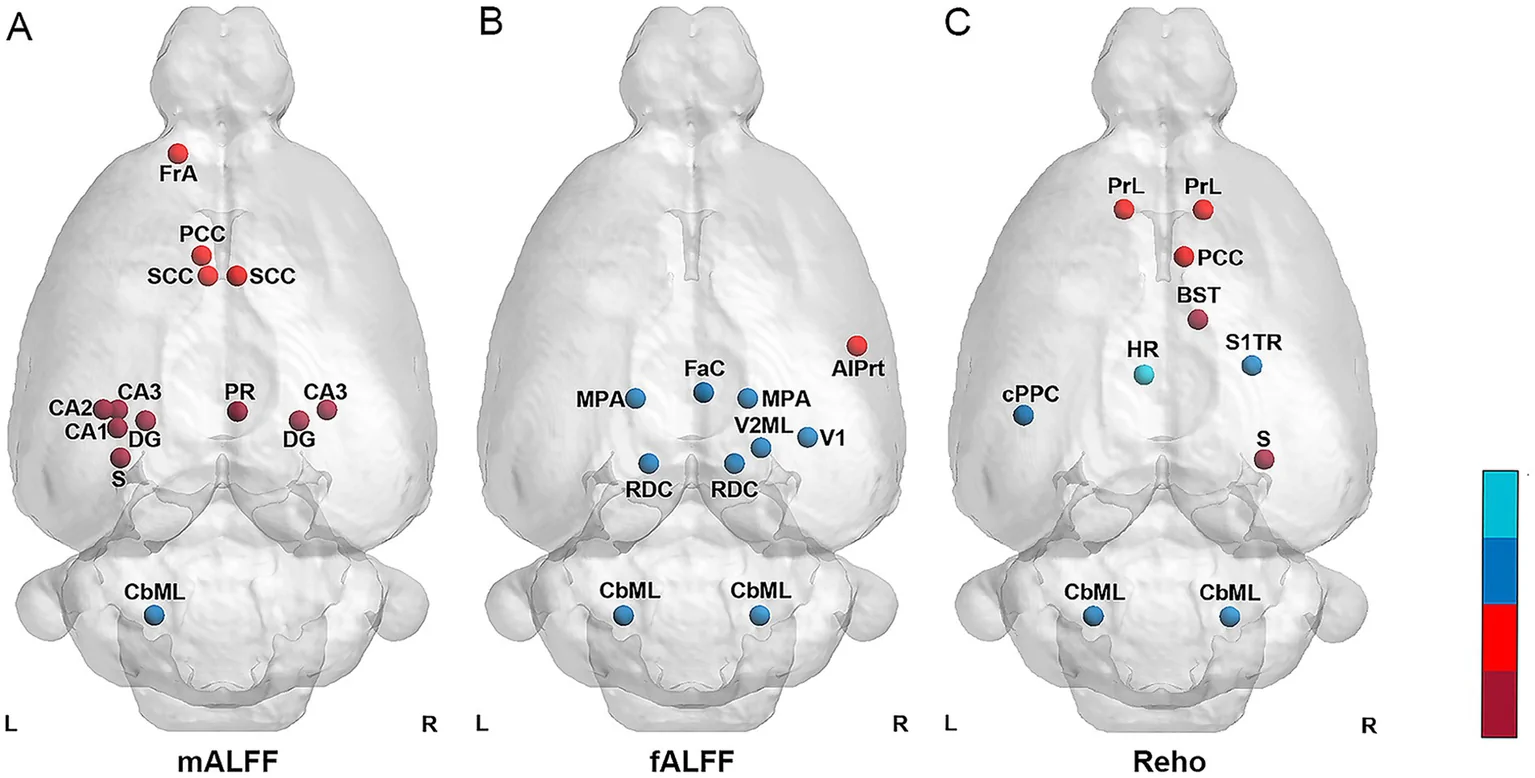
Characteristics of regional neural activity in functional brain networks. Whole-brain surface projections illustrate significant alterations in intrinsic regional activity characterized by **(A)** mean amplitude of low-frequency fluctuation (mALFF), **(B)** fractional amplitude of low-frequency fluctuation (fALFF), and **(C)** mean regional homogeneity (mReHo) (*p* < 0.05, FDR corrected). Group differences are represented by color-coded nodes: Dark red nodes signify regions significantly increased in the BCCAO group compared to the SHAM group. Red nodes signify regions significantly decreased in the BCCAO group compared to the SHAM group. Dark blue nodes signify regions significantly increased in the CCYC group compared to the BCCAO group. Light blue nodes signify regions significantly decreased in the CCYC group compared to the BCCAO group. Specific anatomical coordinates and statistical values are demonstrated in Supplementary Table S2. AIPrt, Posterior Agranular Insular Cortex; BST, Bed Nucleus of the Stria Terminalis; CA1, Cornu Ammonis 1; CA2, Cornu Ammonis 2; CA3, Cornu Ammonis 3; CbML, Molecular Layer of the Cerebellum; PCC, Primary Cingular Cortex; SCC, Secondary Cingular Cortex; DG, Dentate Gyrus; FaC, Fasciola Cinereum; FrA, Frontal Association Cortex; HR, Hypothalamic Region; MPA, Medial Parietal Associative Cortex; OB, Olfactory Bulb; PR, Pretectal Region; cPPC, Parietal Cortex Postero Caudal Part; PrL, PreLimbic System; RDC, Retrosplenial Dysgranular Cortex; S1TR, Primary Somatosensory Cortex Trunk; S, Subiculum; V1, Primary Visual Cortex; V2ML, Medio Lateral Secondary Visual Cortex.

We next examined regional neural activity changes using mALFF, fALFF, and mReHo. Three metrics were calculated to describe the regional neural activity, summarized in Figure 3. Detailed results are shown in Supplementary Table S2.

Compared with the SHAM group, mALFF of hippocampus formation (HF) was increased in the BCCAO group, mALFF of the limbic system and cingular system was significantly decreased, see Figure 3A. fALFF of posterior agranular insular cortex-R (AIPrt-R) of the insular system in the BCCAO group was significantly lower than the SHAM group, see Figure 3B. Additionally, mReHo of the HF and the thalamus were significantly increased in the BCCAO group, and the limbic system, the cingular system and the insular system in the BCCAO group were significantly lower than the SHAM group, see Figure 3C.

Compared with the BCCAO group, fALFF of the HF, retrosplenial system and the parietal system in the CCYC group were significantly higher than the BCCAO group, see Figure 3B. Additionally, compared with the BCCAO group, mReHo of the parietal system, the somatosensory system and the sensory-motor system in the CCYC group were significantly increased. Meanwhile, mReHo of the hypothalamus in the CCYC group was significantly lower than the BCCAO group, see Figure 3C.

### 3.4 Association between Regional brain activity and learning and memory ability

Spearman correlation analyses were performed between MWM behavioral indices and regional functional metrics, partly shown in Figure 2. Detailed results are shown in Supplementary Table S3.

Escape latency was negatively correlated with functional metrics in the frontal and insular regions, including mALFF in FrA-L, fALFF in AIPrt-R, and mReHo in agranular dysgranular insular cortex-R (AID-R), see Figures 2C–E. Crossing number was positively correlated with functional metrics in the insular, retrosplenial, cingulate, and somatosensory regions, including fALFF in AID-R and retrosplenial dysgranular cortex-L (RDC-L) and mReHo in primary cingular cortex-R (PCC-R) and secondary somatosensory cortex-R (S2-R), see Figures 2G–I. The time in target quadrant was positively correlated with functional metrics in the insular system, retrosplenial cortex, hippocampal formation, and the pallidum, including fALFF in AID-R, RDC-L, and bilateral subiculum, as well as with mReHo in globus pallidus-L (GP-L), see Figures 2K–M.

### 3.5 CCYC-related changes in nodal topological properties

**Figure 4:**
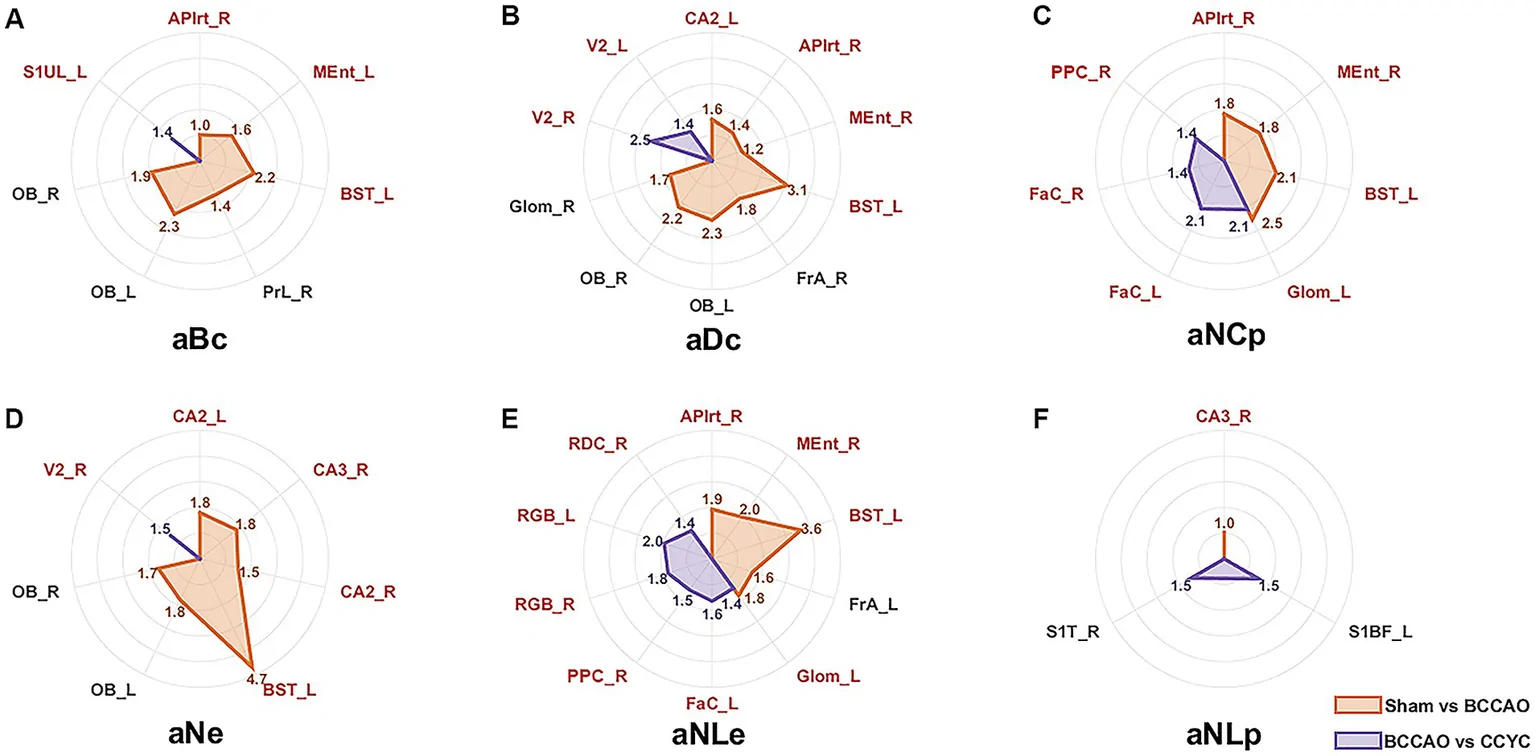
Effects of CCYC on nodal topological properties of the brain functional network in VaD rats. Radar charts represent the |Cohen’s *d*| values for six key nodal metrics: **(A)** area betweenness centrality (aBc), **(B)** area degree centrality (aDc), **(C)** area nodal clustering coefficient (aNCp), **(D)** area nodal efficiency (aNe), **(E)** area nodal local efficiency (aNLe), and **(F)** area nodal shortest path length (aNLp). The orange area indicates the effect size of differences between the SHAM and BCCAO groups. The purple area indicates the effect size of differences between the BCCAO and CCYC groups. Red and black labels, respectively, denote brain regions with positive and negative Cohen’s *d* values. AIPrt, Posterior Agranular Insular Cortex; BST, Bed Nucleus of the Stria Terminalis; CA1, Cornu Ammonis 1; CA2, Cornu Ammonis 2; CA3, Cornu Ammonis 3; CbML, Molecular Layer of the Cerebellum; PCC, Primary Cingular Cortex; FaC, Fasciola Cinereum; FrA, Frontal Association Cortex; Glom, Glomerular Layer of the Accessory Olfactory Bulb; MEnt, Medial Entorhinal Cortex; OB, Olfactory Bulb; PrL, PreLimbic System; RDC, Retrosplenial Dysgranular Cortex; RGB, Retrosplenial Granular Cortex Part B; S1BF, Primary Somatosensory Cortex Barrel field; S1T, Primary Somatosensory Cortex Trunk; S1UL, Primary Somatosensory Cortex Upperlips; V2, Secondary Visual Cortex.

To further characterize network-level alterations, we examined nodal topological metrics across the 158 ROI-based functional network. The overall patterns are summarized in Figure 4, and detailed results are provided in Supplementary Table S4.

Nodal metrics included area betweenness centrality (aBc), area degree centrality (aDc), area nodal clustering coefficient (aNCp), area nodal efficiency (aNe), area nodal local efficiency (aNLe), and area nodal shortest path length (aNLp).aBc and aDc are node centrality metrics, measuring the importance of a node as a “traffic hub” or “information bridge” (25). aNe, aNLe, and aNCp are node efficiency metrics, measuring the “local information processing efficiency” of a node and its neighboring regions. aNLp measures the “average path distance” from a node to all other nodes in the brain. Consistent with global metrics, most comparisons of nodal metrics did not survive strict *FDR* correction (*P_FDR* > 0.05). However, all comparisons consistently demonstrated substantial effect sizes (|Cohen’s *d*| > 1.0, with some exceeding 3.0 or 4.0), indicating the presence of strong statistical trends. Notably, the aNLp metric reached statistical significance after *FDR* correction in some regions.

Compared with the SHAM group, the BCCAO group showed reduced nodal centrality and efficiency (aBc, aDc, and aNLe) in anterior cortical regions, particularly the frontal association cortex (FrA) and prelimbic system, see Figure 4, orange profiles. In contrast, increased nodal measures (aBc, aDc, aNCp, aNe, aNLe) were observed in several subcortical and medial temporal regions, including the bed nucleus of the stria terminalis (BST), hippocampal CA2/CA3, amygdalopiriform complex (APir), and entorhinal cortex, together with prolonged nodal shortest path length in some regions. The medial entorhinal cortex (MEnt) showed broadly elevated nodal metrics, whereas the olfactory bulb (OB) and related regions showed reduced centrality-related measures (aBc, aDc, and aNe). See Figure 4 and Supplementary Table S4.

Compared with the BCCAO group, the CCYC group showed increased nodal centrality and efficiency in the retrosplenial-cingulate system (aNCp, aNLe), hippocampal system (aNCp, aNLe), visual system (aDc, aNe), and somatosensory system (aBc). Retrosplenial system (NLe) were significantly enhanced in the CCYC group, see Figure 4, purple profiles. Among these findings, the primary somatosensory cortex barrel field (S1BF) showed increased aBc and reduced aNLp that survived FDR correction. The remaining nodal differences are presented as exploratory results together with their effect sizes. See Figure 4 and Supplementary Table S4.

### 3.6 Impact of CCYC on full-brain functional connectivity

**Figure 5:**
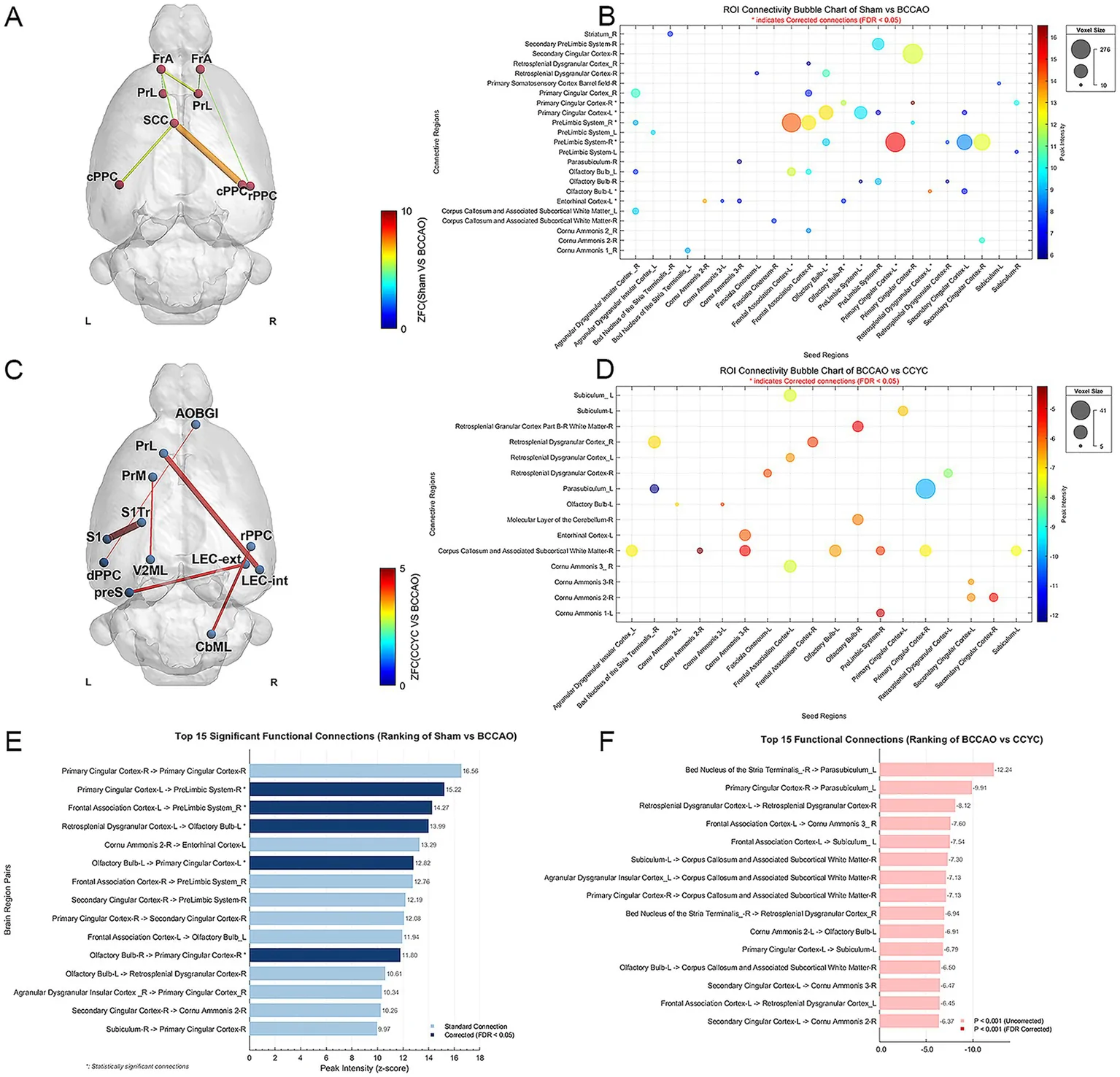
Characterization of whole-brain and ROI-based functional connectivity (FC). Functional network visualizations and quantitative analyses illustrate significant alterations and restorative trends in connectivity. **(A)** Network-based statistic (NBS) analyses illustrate significantly decreased FC in the BCCAO group compared to the SHAM group (*p* < 0.05, NBS corrected). Nodes are color-coded by functional systems, and edges represent functional connection strength. **(B)** ROI-based functional connectivity alterations using seed points for the BCCAO group compared to the SHAM group (*p* < 0.05, FDR corrected). **(C)** NBS analyses illustrate an enhanced connectivity trend in the CCYC group compared to the BCCAO group (*p* < 0.001, uncorrected). Nodes are color-coded by functional systems, and edges represent functional connection strength. **(D)** ROI-based functional connectivity alterations using seed points for the CCYC group compared to the BCCAO group (*p* < 0.001, uncorrected). In bubble plots, the *x*-axis represents the selected seeds and the *y*-axis represents the ROIs showing significant connectivity with these seeds; sphere size represents the number of voxels, and sphere color represents the peak intensity (redder spheres signify higher values). Ranking of the top 15 strongest functional connections based on ROI-FC analyses for **(E)** SHAM vs. BCCAO groups and **(F)** BCCAO vs. CCYC groups. Dark blue bars represent connections that remained significant after statistical correction. Specific anatomical regions and statistical values are demonstrated in Supplementary Tables S6. AOBGl, Glomerular Layer of the Accessory Olfactory Bulb; CbML, Molecular Layer of the Cerebellum; cPPC, Parietal Cortex Postero Caudal Part; dPPC, Parietal Cortex Postero Dorsal Part; FrA, Frontal Association Cortex; LEC-ext, Lateral Entorhinal Cortex external part; LEC-int, Lateral Entorhinal Cortex Internal part; preS, preSubiculum; PrL, PreLimbic System; PrM, Primary Motor Cortex; rPPC, Parietal Cortex Postero Rostral; S1, Primary somatosensory cortex; S1Tr, Primary Somatosensory Cortex Trunk; SCC, Secondary Cingular Cortex; V2ML, Medio Lateral Secondary Visual Cortex.

Whole-brain functional connectivity was analyzed using network-based statistics (*NBS*). The overall patterns are summarized in Figures 5A,C, and detailed results are provided in Supplementary Table S5. Compared with the SHAM group, the BCCAO group showed a significant subnetwork with reduced functional connectivity (*NBS*, *p* < 0.05), mainly involving connections between the limbic system (FrA/prelimbic system) and cingulate gyrus system, as well as within these systems. See Figure 5A.

No significant subnetwork was detected between CCYC and the BCCAO groups after *NBS* correction. However, at an exploratory threshold (*p* < 0.001, uncorrected), the CCYC group showed higher connectivity than the BCCAO group in several connections, including those between the lateral entorhinal cortex (LEC) and prelimbic cortex (PrL), between the LEC and presubiculum, between the cerebellar molecular layer (CbML) and posterior rostral parietal cortex (rPPC), and between the Medio lateral secondary visual cortex (V2ML) and primary motor cortex (PrM), as well as within the somatosensory system. See Figure 5C.

### 3.7 Impact of CCYC on functional connectivity based on ROI

Seed-based functional connectivity analyses were performed using the frontal association cortex (FrA), prelimbic cortex (PrL), retrosplenial cortex (RSC), and primary cingulate cortex (PCC) as seed regions. The overall patterns are summarized in Figure 5B,D–F, and detailed results are provided in Supplementary Table S6.

In a word, the BCCAO group exhibited significant, *FDR*-corrected reductions in connectivity, see Figures 5B,E. Specifically, connectivity between the FrA and the contralateral PrL as well as the RDC was significantly disrupted; connectivity between the PrL and the PCC as well as the OB was significantly weakened; connectivity between the RSC and the PrL as well as the OB was also markedly reduced.

In exploratory analyses (*p* < 0.001, uncorrected), the CCYC group exhibited enhanced functional connectivity compared with the BCCAO group between the FrA and the hippocampal CA3 subfield, subiculum, and RSC, see Figures 5D,F. Connectivity between the PCC and the subiculum also exhibited an increasing trend. Meanwhile, multi-seed analyses consistently revealed enhanced functional connectivity with the corpus callosum and associated subcortical white matter (CC-WM) regions in the CCYC group, see Figures 5D,F.

## 4 Discussion

CCYC has been incorporated into Chinese expert guidance for vascular cognitive impairment, yet previous studies have largely focused on therapeutic efficacy and target-level mechanisms rather than systems-level phenotypic alterations in the brain. Using rs-fMRI to characterize the neuroimaging phenotype of VaD as a network disconnection disorder, we found that BCCAO-induced injury was centered on the fronto-retrosplenial-hippocampal memory circuit, and that CCYC was associated with improved spatial memory, partial remodeling of this circuit, and broader changes in whole-brain functional organization.

### 4.1 The fronto-retrosplenial-hippocampal pattern characterizes regional, connectivity, and nodal network abnormalities in BCCAO

Regional, connectivity, and nodal analyses consistently indicated that BCCAO did not induce scattered functional abnormalities, but rather produced a structured pattern centered on frontal, retrosplenial, and hippocampal regions. At the regional level, BCCAO rats showed reduced mALFF and mReHo in frontal-cingulate regions, together with increased activity in the hippocampal system. In contrast, CCYC-associated regional changes were mainly concentrated in posterior and sensory-related systems, including increased fALFF in the hippocampal system, subiculum, RSC, parietal cortex, cerebellar molecular layer (CbML), together with increased mReHo in the parietal cortex, somatosensory cortex, and CbML.

To further determine whether the observed regional neural alterations were behaviorally relevant, we examined the relationships between Morris Water Maze performance and regional neural activity. Behavioral correlation analyses showed that Morris water maze performance was associated with regional neural activity in several areas, including the FrA, RSC, subiculum, insula, and S2. Specifically, escape latency was negatively correlated with mALFF in the left FrA. Crossing number was positively correlated with fALFF in the RSC. Time spent in the target quadrant was positively correlated with mReHo in the subiculum. In addition, neural activity in the insula and S2 was significantly associated with MWM performance.

At the circuit level, whole-brain functional connectivity analysis showed a broad reduction in coupling in BCCAO rats, particularly involving anterior–posterior interactions. ROI-based analyses further localized this pattern to reduced connectivity between the frontal association cortex and contralateral prelimbic/retrosplenial regions, between the prelimbic cortex and cingulate/olfactory regions, and between the retrosplenial cortex and prelimbic/olfactory regions. Compared with the BCCAO group, CCYC treatment was associated with exploratory increases in connectivity, mainly involving frontal-hippocampal/subicular and retrosplenial-subicular pathways. Increased connectivity was also observed between cortical regions and corpus callosum/subcortical white matter-related regions.

Nodal graph analysis further complemented the functional connectivity findings by revealing region-specific alterations in hub organization and network efficiency. At the nodal level, the BCCAO group showed a pronounced anterior–posterior dissociation in network topology. Reductions in nodal centrality- and efficiency-related properties were mainly observed in anterior regions, especially the frontal association cortex and anterior limbic system. By contrast, the thalamus, hippocampal system, amygdala, and entorhinal cortex showed large-effect-size increasing trends across multiple nodal measures. Compared with the BCCAO group, CCYC treatment was associated with enhanced nodal centrality and efficiency in posterior default mode and memory-related regions, particularly the retrosplenial-cingulate and hippocampal systems, as well as visual and somatosensory areas.

### 4.2 The behavioral and functional relevance of fronto-retrosplenial-hippocampal remodeling after CCYC treatment

The combined regional, behavioral, and connectivity findings suggest that BCCAO did not simply induce scattered functional abnormalities, but rather produced a structured disturbance centered on the fronto-retrosplenial-hippocampal memory circuit that is highly relevant to spatial cognition. At the regional level, the most prominent pattern in BCCAO rats was a reduction of mALFF and mReHo in frontal-cingulate regions together with increased activity in the hippocampal system and thalamus. This distribution suggests an anterior–posterior imbalance rather than a uniform suppression of brain function, with reduced activity in executive-control regions and heightened activity in hippocampal and subcortical regions. Such a pattern is consistent with previous neuroimaging observations in VaD and vascular cognitive impairment, in which frontal-cingulate dysfunction coexists with abnormal activation of memory-related structures under CCH (6, 10, 26–28). In this context, the CCYC-associated increases in fALFF within the hippocampal system, subiculum, retrosplenial cortex, and parietal cortex are unlikely to represent isolated regional effects. Rather, they point to a selective modulation of the posterior memory-related system that underlies spatial representation and memory processing. At the same time, the increases in parietal, somatosensory, and cerebellar-related regions suggest that this modulation was accompanied by recruitment of auxiliary systems that may support task performance at the systems level (4, 28, 29).

The behavioral correlation results further refine this interpretation by showing that different components of MWM performance mapped onto different nodes within the same functional axis. Escape latency was negatively correlated with mALFF in the left FrA, whereas crossing number and time spent in the target quadrant were positively correlated with activity in the RSC and subiculum, respectively. Frontal regions, including the frontal association cortex, are more closely related to executive control, strategy selection, and the organization of behavior during navigation, whereas the retrosplenial cortex and hippocampal formation are more directly involved in spatial representation, contextual integration, and memory retention (27, 28, 30). The subiculum is especially important in this framework because it is a major output relay of the hippocampal formation and an essential interface through which hippocampal information is transmitted to broader cortical systems involved in navigation and memory retrieval (27, 28, 31). Therefore, the correlation pattern observed here suggests a functional division of labor within the affected network: frontal dysfunction was more closely linked to impaired learning efficiency, whereas retrosplenial and subicular dysfunction was more closely linked to impaired spatial memory retention. The additional correlations involving the insula and S2 further suggest that sensory-integrative regions may participate when the core memory-related system is compromised, but these auxiliary regions appear to play a supporting rather than primary role (4).

The functional connectivity findings extend this argument from the level of regional abnormalities to the level of circuit organization. In BCCAO rats, both whole-brain and ROI-based analyses showed reduced coupling between anterior and posterior parts of the network, particularly between prefrontal/cingulate regions and retrosplenial-hippocampal regions. Similar patterns of disrupted functional connectivity have also been reported in subcortical vascular cognitive impairment and related vascular cognitive disorders in resting-state fMRI studies (32, 33). These findings indicate that the abnormalities identified by ALFF and ReHo were not merely local disturbances, but were embedded in a broader failure of communication between executive-control regions and posterior memory-related systems. This interpretation accords with previous reports that disruption of large-scale anterior–posterior functional coupling is closely linked to cognitive decline in VaD (26). Against this background, the CCYC-associated increases in connectivity are particularly informative. Although several comparisons remained exploratory, the pattern of increased coupling was not random: it was concentrated in frontal-hippocampal/subicular, retrosplenial-subicular, and cortical–subcortical pathways. The frontal cortex contributes to executive control and strategy selection during navigation tasks, while the RSC serves as an important gateway linking cortical cognitive networks with hippocampal memory systems (especially the subiculum), and contributes to the integration of spatial representations and contextual information (27, 28). Particularly, the subiculum, as the major output relay of the hippocampal formation, is essential for transmitting hippocampal spatial information to cortical regions involved in memory retrieval and navigation (27, 28, 31). Through this integration of executive control, spatial representation, and memory encoding, these regions constitute the fronto-retrosplenial-hippocampal memory circuit that supports higher-order cognitive processing (26, 30). Thus, when the regional, behavioral, and connectivity findings are considered together, the most coherent interpretation is that CCYC did not simply “activate more regions,” but instead promoted partial reorganization of the disrupted fronto-retrosplenial-hippocampal memory circuit that is centrally involved in spatial cognition (26–28, 30, 31). The additional enhancement of cortical–subcortical white matter-related connectivity further suggests that this reorganization may extend beyond the core memory circuit and may help re-establish broader network integration under chronic hypoperfusion.

### 4.3 The chemical profile of CCYC is consistent with multi-level protection against network disconnection

These imaging findings suggest that the protective effects of CCYC are not confined to isolated brain regions, but may instead reflect interference with the CCH-driven pathological cascade that underlies brain disconnection in VaD. Prolonged CCH induces neurovascular dysfunction, oxidative stress, neuroinflammation, and white matter injury, ultimately disrupting long-range connectivity and contributing to “disconnection syndrome” (3–5).

Against this background, the chemical profile of CCYC might be relevant to support the view of its brain-network modulation. Acteoside and nuciferine may preserve microvascular and neurovascular unit function (34, 35). Echinacoside and emodin may act on oxidative stress, inflammatory responses, and ischemia-related injury triggered by CCH (36, 37). Quercetin and eicosapentaenoic acid may be particularly relevant to the protection of white matter and demyelinating injury (38, 39). This integrated mechanism may help explain why CCYC in the present study produced improvements within the fronto-retrosplenial-hippocampal memory circuit rather than isolated local effects.

Among these possibilities, improvement of hypoperfusion-related microvascular dysfunction may be particularly relevant to the BCCAO model. This interpretation is consistent with our previous finding that CCYC alleviated chronic hypoxia-related microvascular injury and regulated HIF-1α- and VEGF-related signaling (16). However, these findings provide only indirect support for the present imaging results.

## 5 Conclusion

CCYC has been widely used clinically for vascular cognitive disorders, but target- and pathway-based analyses alone are insufficient to explain how multi-component Chinese patent medicines influence brain function. Using rs-fMRI combined with behavioral assessment, we found that VaD in BCCAO rats was characterized by abnormalities in the frontal association cortex, retrosplenial cortex, and hippocampal system, involving regional activity, inter-regional coupling, and nodal topology. CCYC was associated with improved spatial cognition and partial remodeling of these abnormalities within the fronto-retrosplenial-hippocampal memory circuit, accompanied by broader topological reorganization.

## 6 Limitations

Several limitations should be acknowledged. First, the study included neither diffusion MRI/tractography nor myelin- or axon-specific histology. Thus, “disconnection syndrome” refers to reduced BOLD-based coupling and altered network topology, not anatomically verified white matter tract disruption. Likewise, BOLD correlations involving atlas-defined corpus callosum and white matter ROIs do not demonstrate tract integrity, axonal preservation, or remyelination. Second, rs-fMRI lacks molecular specificity, and BOLD signals depend partly on neurovascular coupling; therefore, the observed changes cannot distinguish neuronal recoupling from altered vascular responsiveness. Future studies should integrate diffusion MRI, histological validation, cerebral perfusion measurements, and pathway-specific interventions. Additionally, while pooled correlation analyses across all experimental groups provided an overall trend between behavioral and imaging markers, the clustering of observations by group suggests these correlations may partially reflect between-group intervention effects alongside within-group biological associations.

## Data Availability

The original contributions presented in the study are included in the article/Supplementary material, further inquiries can be directed to the corresponding author.

## Footnotes

1
https://github.com/rordenlab/dcm2niix

2
http://www.fil.ion.ucl.ac.uk/spm

3
http://rfmri.org/dpabi

4
https://www.nitrc.org/projects/nbs

5
http://www.nitrc.org/projects/gretna

## Glossary

VaD: Vascular Dementia
VCI: Vascular Cognitive Impairment
CCH: Chronic Cerebral Hypoperfusion
AD: Alzheimer’s disease
CCYC: Compound Congrong Yizhi Capsule
BCCAO: Bilateral common carotid artery occlusion
rs-fMRI: Resting-state functional Magnetic Resonance Imaging
BOLD: Blood-Oxygenation-Level-Dependent
ALFF: Amplitude of low-frequency fluctuations
mALFF: Mean Amplitude low-frequency fluctuations
fALFF: Fractional Amplitude of low-frequency fluctuations
ReHo: Regional homogeneity
mReHo: Mean Regional homogeneity
FC: Functional connectivity
ROI-FC: Region of Interest-based Functional Connectivity
DMN: Default Mode Network
SFC: Structure–Function Coupling
ROI: Region of Interest
aBc: Area betweenness centrality
aDc: Area degree centrality
aNe: Area nodal efficiency
aNLe: Area nodal local efficiency
aNCp: Area nodal clustering-coefficient
aNLp: Area nodal shortest path length
MWM: Morris Water Maze
NBS: Network-Based Statistic
FDR: False Discovery Rate
AUC: Area Under the Curve
Cohen’s *d*: Effect size measure
HF: Hippocampus formation
CA1/CA2/CA3: Cornu Ammonis regions 1/2/3
DG: Dentate Gyrus
S/Sub: Subiculum
PCC: Primary Cingular Cortex
SCC: Secondary Cingular Cortex
RSC: Retrosplenial Cortex
RDC/RGB: Retrosplenial Dysgranular/Granular
FrA: Frontal Association Cortex
PFC: Prefrontal Cortex
PrL: PreLimbic System/Cortex
OB: Olfactory Bulb
AOBGl: Accessory Olfactory Bulb, Glomerular Layer
MEnt/LEC: Medial/Lateral Entorhinal Cortex
AIPrt/AID: Posterior/Dysgranular Insular Cortex
S1/S2: Primary/Secondary Somatosensory Cortex
S1BF/S1TR: S1 Barrel field/Trunk
V1/V2: Primary/Secondary Visual Cortex
V2ML/V2IP: V2 Medio Lateral/Intermediate Posterior
cPPC/dPPC/rPPC: Parietal Cortex (Caudal/Dorsal/Rostral)
MPA: Medial Parietal Associative Cortex
PrM: Primary Motor Cortex
CbML: Molecular Layer of the Cerebellum
BST: Bed Nucleus of the Stria Terminalis
GP-L: Globus pallidus-Left
FaC: Fasciola Cinereum
CC-WM: Corpus Callosum and subcortical White Matter
