# Supplementary material for: Compound Congrong Yizhi capsule partially remodels the fronto-retrosplenial-hippocampal memory circuit in vascular dementia rats
Source: Front Neurol. 2026 Sep 1;17:1867157. doi: 10.3389/fneur.2026.1867157 (PMC13574697; doi:10.3389/fneur.2026.1867157)
Supplement: Supplementary file 1 [file Table_1.DOCX]

Supplementary Material

# 1 Supplementary Data

## 1.1 Supplementary Methods S1. Detailed chemical characterization of CCYC

Chemical profiling was performed on a DIONEX Ultimate 3000 UPLC system coupled to a Thermo Q Exactive mass spectrometer. Chromatographic separation was achieved on a Thermo Hypersil Gold C18 column (3 μm, 2.1 × 100 mm). The mobile phase consisted of 2 mmol/L ammonium formate with 0.1% formic acid in water and acetonitrile. The flow rate was 0.25 mL/min and the injection volume was 5 μL. The gradient program was as follows: 95% aqueous phase for 2 min, linearly decreased to 5% over 40 min, maintained for 5 min, and then returned to the initial condition over 3 min.

Mass spectrometry was conducted in positive and negative electrospray ionization modes. The spray voltage was set at +3500 V and -2800 V, with capillary and evaporation temperatures of 320 °C and 350 °C, respectively. Sheath gas and auxiliary gas were set at 40 and 10 arbitrary units. Full-scan MS data were acquired over a mass range of 100–1500 m/z at a resolution of 70,000, followed by data-dependent MS/MS scans at a resolution of 35,000 with stepped normalized collision energies of 20, 40, and 60.

## 1.2 Supplementary Methods S2. Detailed MRI acquisition parameters

MRI scans were performed on a 9.4 T animal MRI scanner using a volume transmit coil and a surface receive coil.

High-resolution T2-weighted anatomical images were acquired using a turboRARE sequence with the following parameters: TR = 14697 ms, TE = 33 ms, echo spacing = 11 ms, RARE factor = 8, 140 axial slices, slice thickness = 0.2 mm, no gap, field of view = 18 × 23 mm, matrix size = 90 × 115, spatial resolution = 0.2 × 0.2 × 0.2 mm³, and 4 averages.

Resting-state BOLD-fMRI data were acquired using a multi-slice gradient-echo EPI sequence with the following parameters: TR = 3000 ms, TE = 6.838 ms, flip angle = 30°, matrix size = 107 × 90, field of view = 32 × 27 mm², 73 slices, slice thickness = 0.30 mm, no gap, bandwidth = 500,000 Hz, 140 volumes, and spatial resolution = 0.30 × 0.30 × 0.30 mm³.

## 1.3 Supplementary Methods S3. Detailed image preprocessing

Raw DICOM images were converted to NIfTI format using dcm2niix [27]. All images were scaled by a factor of 10 by adjusting voxel size information in the image header without interpolation, thereby preserving the original image data. Except for internal optimization during motion correction, the subsequent preprocessing steps were scale-invariant.

Resting-state fMRI data were preprocessed in SPM12 [28,29]. To allow signal stabilization, the first five volumes of each functional scan were discarded. The remaining images underwent intensity normalization, slice-timing correction, motion realignment, and co-registration. After resetting the image origin to the anterior commissure, images were spatially normalized to the SIGMA EPI Brain Template aligned with the Paxinos and Watson rat brain atlas [30,31] using a non-linear transformation, and resampled to 0.3 × 0.3 × 0.3 mm³. The normalized images were then spatially smoothed with a Gaussian kernel of 0.6 mm full-width at half-maximum. Finally, linear detrending and temporal band-pass filtering (0.01–0.1 Hz) were applied to reduce low-frequency drift and high-frequency physiological noise.

## 1.4 Supplementary Methods S4. Detailed regional rs-fMRI metric calculation

Regional rs-fMRI metrics, including ALFF, fALFF, and ReHo, were first computed voxel-wise across the whole brain. The brain was then parcellated into 158 regions of interest (ROIs), and mean regional values were extracted from each ROI.

For each ROI, the BOLD time series was averaged across all constituent voxels. Fast Fourier transform (FFT) was then applied to the mean time series to obtain the power spectrum. ALFF was calculated as the sum of amplitudes within the low-frequency band (0.01–0.08 Hz). fALFF was calculated as the ratio of power within the low-frequency band to power across the entire detectable frequency range (e.g., 0.01–0.25 Hz). Mean ALFF and fALFF values for each ROI were extracted from the corresponding voxel-wise maps.

For ReHo, Kendall’s coefficient of concordance (Kendall’s W) was calculated between each voxel and its 26 nearest neighbors to generate a whole-brain ReHo map. Each voxel value was normalized by the whole-brain mean ReHo value, and the mean normalized ReHo value within each ROI was extracted to generate the final mReHo values.

Group differences in mALFF, mfALFF, and mReHo were assessed using two-sample t-tests in SPSS 23.0, with false discovery rate (FDR) correction for multiple comparisons across the 158 ROIs (*P-FDR* < 0.05).

## 1.5 Supplementary Methods S5. Detailed functional connectivity analysis

For ROI-to-ROI functional connectivity, atlas-defined ROI masks were transformed from template space to native functional space using the inverse normalization deformation field. Mean BOLD time series were extracted from each ROI, and a 158 × 158 connectivity matrix was generated by calculating Pearson correlation coefficients between all ROI pairs. Correlation coefficients were transformed to z scores using Fisher’s r-to-z transformation.

For seed-based functional connectivity, Pearson correlation coefficients were calculated between the mean time series of a selected seed ROI and all other brain voxels. Correlation maps were then transformed to z-score maps for group-level analysis.

## 1.6 Supplementary Methods S6. Detailed graph theoretical analysis

The 158 ROIs were defined as network nodes, and ROI-to-ROI correlations were used as edges. Absolute z values were used to generate unsigned weighted connectivity matrices. Networks were constructed across a *sparsity* range of 0.05–0.40 in steps of 0.02. Both weighted and binary networks were generated at each threshold.

Nodal metrics included area betweenness centrality (aBc), area degree centrality (aDc), area nodal clustering coefficient (aNCp), area nodal efficiency (aNe), area nodal local efficiency (aNLe), and area nodal shortest path length (aNLp).

To obtain threshold-independent summary measures, the area under the curve (AUC) was calculated for each metric across the full sparsity range using the trapezoidal rule. Subjects with disconnected networks at any threshold were excluded from graph-theoretical quality control.

The 158 ROIs were defined as network nodes, and Fisher’s z-transformed Pearson correlation coefficients between all ROI pairs were used as edges. Absolute z values were used to generate matrices of unsigned connection weights. Brain networks were constructed across a *sparsity* range of 0.05–0.40 in increments of 0.02. Both weighted and binary networks were generated at each sparsity threshold, and global and nodal metrics were calculated for each subject at each threshold.

To obtain threshold-independent summary measures, the area under the curve (AUC) was calculated for each metric across the full sparsity range using the trapezoidal rule:

$$\mathrm{AUC} = \sum_{i=1}^{n-1} \frac{M\left( S_{i} \right) + M\left( S_{i+1} \right)}{2} \left( S_{i+1} - S_{i} \right)$$

Quality control was performed by excluding any subject whose brain network was not fully connected at any of the applied thresholds. Group differences in AUC values were assessed using two-sample t-tests with *FDR* correction. Effect sizes (*Cohen’s d*) were additionally calculated for exploratory interpretation; positive values indicated higher values in the first group and negative values indicated higher values in the second group.

# 2 Supplementary Tables

## 2.1 Supplementary table S1. Identification of chemical constituents in CCYC by LC-MS/MS.

| **Components** | **Molecular Formula** | **Ionization Mode** | **Exact Mass (m/z）** | **Tandem Mass Spectrometry** | **Retention Times (min)** |
| --- | --- | --- | --- | --- | --- |
| Arachidonate | C20H32O2 | M-H | 303.23295 | 303.23236 | 34.42 |
| Suchilactone | C21H20O6 | M+H | 369.13326 | 277.21631 | 24.84 |
| Quercetin | C15H10O7 | M+H | 303.04992 | 303.05005 | 14.07 |
| Echinacoside | C35H46O20 | M-H | 785.25096 | 161.02306 | 8.92 |
| Acteoside | C29H36O15 | M-H | 623.19814 | 161.02299 | 11.06 |
| Physcion | C16H12O5 | M-H | 283.06119 | 240.04192 | 19.53 |
| Emodin | C15H10O5 | M-H | 269.04554 | 225.05490 | 23.25 |
| Citreorosein | C15H10O6 | M-H | 285.04046 | 257.04468 | 25.00 |
| Questinol | C16H12O6 | M+H | 301.07066 | 283.06015 | 19.24 |
| Liquiritigenin | C15H12O4 | M+H | 257.08083 | 239.06999 | 10.13 |
| Liquiritin | C21H22O9 | M-H | 419.13365 | 255.06522 | 10.75 |
| Eicosapentaenoic acid | C20H30O2 | M-H | 301.21730 | 301.21716 | 32.49 |
| Linolenic acid | C18H30O2 | M+H | 279.23185 | 149.02348 | 23.08 |
| Myristic acid | C14H28O2 | M-H | 227.20165 | 227.20081 | 46.35 |
| Cycloartenol | C30H50O | M+H | 427.39344 | 237.12721 | 42.18 |
| Remerin | C18H17NO2 | M+H | 280.13320 | 249.09088 | 13.40 |
| Nuciferin | C19H21NO2 | M+H | 296.16450 | 265.12219 | 13.36 |
| O-Nornuciferine | C18H19NO2 | M+H | 282.14885 | 191.08553 | 10.75 |
| Isorhamnetin | C16H12O7 | M+H | 317.06557 | 317.06567 | 18.29 |
| Armepavine | C19H23NO3 | M+H | 314.17507 | 107.04958 | 9.27 |
| Kaempferol | C15H10O6 | M+H | 287.05501 | 269.04434 | 14.09 |
| Machiline | C17H19NO3 | M+H | 286.14377 | 107.04958 | 7.68 |
| (1S)-1-(4-hydroxybenzyl)-2-methyl-3,4-dihydro-1H-isoquinoline-6,7-diol | C17H19NO3 | M+H | 286.14377 | 107.04958 | 9.99 |

## 2.2 Supplementary table S2. Resting-State Functional Characteristics of Brain Regions between groups

| **Comparision** | **Trend** | **System** | **Regions** | **Side** | **CohenD** | ***P-fdr*** |
| --- | --- | --- | --- | --- | --- | --- |
| ***mALFF*** |  |  |  |  |  |  |
| **Sham vs Model** | **Increased** | Hippocampus Fomation | Cornu Ammonis 2 | L | -3.0624 | 0.0029 |
|  |  |  | Subiculum | L | -2.3020 | 0.0068 |
|  |  |  | Dentate Gyrus | L | -2.3012 | 0.0082 |
|  |  |  | Cornu Ammonis 1 | L | -2.1514 | 0.0154 |
|  |  |  | Cornu Ammonis 3 | R | -2.1677 | 0.0163 |
|  |  |  | Cornu Ammonis 3 | L | -1.9115 | 0.0217 |
|  |  |  | Dentate Gyrus | R | -1.5945 | 0.0452 |
|  |  | Visual System | Pretectal Region | R | -1.7091 | 0.0335 |
|  | **Decreased** | Limbic System | Frontal Association Cortex | L | 1.9532 | 0.0226 |
|  |  | Cingular System | Primary Cingular Cortex | L | 2.3036 | 0.0185 |
|  |  |  | Secondary Cingular Cortex | L | 1.4534 | 0.0257 |
|  |  |  | Secondary Cingular Cortex | R | 1.6348 | 0.0424 |
| **Model vs CCYC** | **Increased** | Sensory-Motor | Molecular Layer of the Cerebellum | L | -1.3675 | 0.0379 |
|  |  |  |  |  |  |  |
| ***fALFF*** |  |  |  |  |  |  |
| **Sham vs Model** | **Decreased** | Insular System | Posterior Agralunar Insular Cortex | R | 2.4623 | 0.0053 |
| **Model vs CCYC** | **Increase** | Hippocampus Fomation | Fasciola Cinereum | R | -1.4563 | 0.0444 |
|  |  | Retrosplenial System | Retrosplenial Dysgranular Cortex | R | -1.8588 | 0.0139 |
|  |  |  | Retrosplenial Dysgranular Cortex | L | -1.9479 | 0.0146 |
|  |  | Parietal System | Medial Parietal Associative Cortex | R | -1.3741 | 0.0429 |
|  |  |  | Medial Parietal Associative Cortex | L | -1.2670 | 0.0492 |
|  |  | Visual System | Medio Lateral Secondary Visual Cortex | R | -1.6170 | 0.0215 |
|  |  |  | Primary Visual Cortex | R | -1.4877 | 0.0377 |
|  |  | Sensory-Motor | Molecular Layer of the Cerebellum | R | -1.6431 | 0.0044 |
|  |  |  | Molecular Layer of the Cerebellum | L | -1.5483 | 0.0096 |
|  |  |  |  |  |  |  |
| ***mReHo*** |  |  |  |  |  |  |
| **Sham vs Model** | **Increased** | Hippocampus Fomation | Subiculum | R | -1.6365 | 0.0242 |
|  |  | Thalamus | Bed Nucleus of the Stria Terminalis | R | -1.5961 | 0.0424 |
|  | **Decreased** | Limbic System | PreLimbic System | L | 2.1708 | 0.0037 |
|  |  |  | PreLimbic System | R | 2.5144 | 0.0051 |
|  |  | Cingular System | Primary Cingular Cortex | R | 2.2090 | 0.0260 |
| **Model vs CCYC** | **Increased** | Parietal System | Parietal Cortex Postero Caudal Part | L | -3.8578 | 0.0021 |
|  |  | Somatosensory System | Primary Somatosensory Cortex Trunk | R | -1.5731 | 0.0313 |
|  |  | Sensory-Motor | Molecular Layer of the Cerebellum | R | -1.5166 | 0.0213 |
|  |  |  | Molecular Layer of the Cerebellum | L | -1.4629 | 0.0354 |
|  | **Decreased** | Hypothalamus | Hypothalamic Region | L | 2.2015 | 0.0086 |

## 2.3 Supplementary table S3. Correlations Between MWM Performance and Regional Brain Activity

| **MWM Metrics** | **Brain Activity** | **System** | **Regions** | **Side** | ***r*** |
| --- | --- | --- | --- | --- | --- |
| Escape Latency | mALFF | Hippocampus Fomation | Subiculum | L | 0.675 |
|  |  | Limbic System | Frontal Association Cortex | L | -0.514 |
|  | mReHo | Hippocampus Fomation | Subiculum | L | 0.609 |
| Crossing Number | fALFF | Retrosplenial System | Retrosplenial Dysgranular Cortex | L | 0.536 |
|  | mReHo | Cingular System | Primary Cingular Cortex | R | 0.544 |
|  |  | Somatosensory System | Secondary Somatosensory Cortex | R | 0.607 |
| Duration in Target Quadrant | fALFF | Retrosplenial System | Retrosplenial Dysgranular Cortex | L | 0.626 |
|  |  | Hippocampus Fomation | Subiculum | L | 0.565 |
|  |  | Hippocampus Fomation | Subiculum | R | 0.521 |

## 2.4 Supplementary table S4. Core Brain Regions Activity Between Groups

| **Comparison** | **Trend** | **System** | **Regions** | **Side** | **CohenD** | ***Pfdr*** |
| --- | --- | --- | --- | --- | --- | --- |
| ***aBc*** |  |  |  |  |  |  |
| **Sham vs Model** | **Increased** | Amygdala | Amygdalopiriform Cortex | R | -1.0178 | 0.5678 |
|  |  | Olfactory System | Medial Entorhinal Cortex | L | -1.5818 | 0.5678 |
|  |  | Thalamus | Bed Nucleus of the Stria Terminalis | L | -2.1614 | 0.4529 |
|  | **Decreased** | Limbic System | PreLimbic System | R | 1.4371 | 0.5678 |
|  |  | Olfactory System | Olfactory Bulb | L | 2.2882 | 0.4582 |
|  |  |  | Olfactory Bulb | R | 1.9269 | 0.5678 |
| **Model vs CCYC** | **Increased** | Somatosensory System | Primary Somatosensory Cortex Upperlips | L | -1.4513 | 0.7446 |
|  |  |  |  |  |  |  |
| ***aDc*** |  |  |  |  |  |  |
| **Sham vs Model** | **Increased** | Hippocampus Formation | Cornu Ammonis 2 | L | -1.6170 | 0.4509 |
|  |  | Amygdala | Amygdalopiriform Cortex | R | -1.3577 | 0.4509 |
|  |  | Olfactory System | Medial Entorhinal Cortex | R | -1.2076 | 0.4509 |
|  |  | Thalamus | Bed Nucleus of the Stria Terminalis | L | -3.0577 | 0.4509 |
|  | **Decreased** | Limbic System | Frontal Association Cortex | R | 1.8084 | 0.4509 |
|  |  | Olfactory System | Olfactory Bulb | L | 2.3043 | 0.4509 |
|  |  |  | Olfactory Bulb | R | 2.2232 | 0.4509 |
|  |  |  | Glomerular Layer of the Accessory Olfactory Bulb | R | 1.7088 | 0.4509 |
| **Model vs CCYC** | **Increased** | Visual System | Medio Lateral Secondary Visual Cortex | R | -2.4900 | 0.8119 |
|  |  |  | Medio Lateral Secondary Visual Cortex | L | -1.4063 | 0.8119 |
|  |  |  |  |  |  |  |
| ***aNCp*** |  |  |  |  |  |  |
| **Sham vs Model** | **Increased** | Amygdala | Amygdalopiriform Cortex | R | -1.8437 | 0.3039 |
|  |  | Olfactory System | Medial Entorhinal Cortex | R | -1.7473 | 0.3039 |
|  |  | Thalamus | Bed Nucleus of the Stria Terminalis | L | -2.0671 | 0.2810 |
|  | **Decreased** | Olfactory System | Glomerular Layer of the Accessory Olfactory Bulb | L | 2.5239 | 0.2633 |
| **Model vs CCYC** | **Increased** | Hippocampus Fomation | Fasciola Cinereum | L | -2.0567 | 0.4859 |
|  |  |  | Fasciola Cinereum | R | -1.4137 | 0.6377 |
|  |  | Cingular System | Secondary Cingular Cortex | R | -1.4133 | 0.6377 |
|  |  | Olfactory System | Glomerular Layer of the Accessory Olfactory Bulb | L | -2.0923 | 0.4859 |
|  |  |  |  |  |  |  |
| ***aNe*** |  |  |  |  |  |  |
| **Sham vs Model** | **Increased** | Hippocampus Fomation | Cornu Ammonis 2 | L | -1.8007 | 0.3544 |
|  |  |  | Cornu Ammonis 3 | R | -1.8178 | 0.4239 |
|  |  |  | Cornu Ammonis 2 | R | -1.5160 | 0.4239 |
|  |  | Thalamus | Bed Nucleus of the Stria Terminalis | L | -4.6771 | 0.3544 |
|  | **Decreased** | Olfactory System | Olfactory Bulb | L | 1.7659 | 0.3544 |
|  |  |  | Olfactory Bulb | R | 1.6557 | 0.4239 |
| **Model vs CCYC** | **Increased** | Visual System | Medio Lateral Secondary Visual Cortex | R | -1.5211 | 0.6218 |
| ***aNLe*** |  |  |  |  |  |  |
| **Sham vs Model** | **Increased** | Amygdala | Amygdalopiriform Cortex | R | -1.9265 | 0.2707 |
|  |  | Olfactory System | Medial Entorhinal Cortex | R | -2.0089 | 0.2483 |
|  |  | Thalamus | Bed Nucleus of the Stria Terminalis | L | -3.6321 | 0.2001 |
|  | **Decreased** | Limbic System | Frontal Association Cortex | L | 1.6534 | 0.2707 |
|  |  | Olfactory System | Glomerular Layer of the Accessory Olfactory Bulb | L | 1.7632 | 0.2001 |
| **Model vs CYCC** | **Increased** | Hippocampus Fomation | Fasciola Cinereum | L | -1.6413 | 0.7896 |
|  |  | Cingular System | Secondary Cingular Cortex | R | -1.4886 | 0.7896 |
|  |  | Retrosplenial System | Retrosplenial Granular Cortex Part B | R | -1.8049 | 0.6162 |
|  |  |  | Retrosplenial Granular Cortex Part B | L | -1.9638 | 0.6162 |
|  |  |  | Retrosplenial Dysgranular Cortex | R | -1.3632 | 0.7896 |
|  |  | Olfactory System | Glomerular Layer of the Accessory Olfactory Bulb | L | -1.4147 | 0.7896 |
|  |  |  |  |  |  |  |
| ***aNLp*** |  |  |  |  |  |  |
| **Sham vs Model** | **Increase** | Hippocampus Fomation | Cornu Ammonis 3 | R | -1.0289 | 0.0252 |
| **Model vs CCYC** | **Decrease** | Somatosensory System | Primary Somatosensory Cortex Barrel field | L | 1.5383 | 0.0370 |
|  |  |  | Primary Somatosensory Cortex Trunk | R | 1.4917 | 0.1024 |

## 2.5 Supplementary table S5. Full-brain Functional Connectivity Between Group (NBS, *P*<0.05)

| **ROI 1** | | **ROI 2** | | **ZFC** |
| --- | --- | --- | --- | --- |
| **System** | **Region** | **System** | **Region** |  |
| **Sham vs Model** | | | | |
| Parietal System | Parietal Cortex Postero Caudal Part-R | Cingular System | Secondary Cingular Cortex-L | 7.2444 |
| Parietal System | Parietal Cortex Postero Caudal Part-L | Cingular System | Secondary Cingular Cortex-L | 5.7044 |
| Limbic System | Frontal Association Cortex-L | Limbic System | PreLimbic System-R | 5.6959 |
| Limbic System | Frontal Association Cortex-L | Cingular System | Secondary Cingular Cortex-L | 5.3584 |
| Limbic System | Frontal Association Cortex-R | Limbic System | PreLimbic System-R | 5.1990 |
| Limbic System | Frontal Association Cortex-R | Parietal System | Parietal Cortex Postero Rostral-R | 5.1601 |
| Limbic System | Frontal Association Cortex-L | Limbic System | PreLimbic System-L | 5.0609 |
| **CCYC vs Model** | | | | |
| Somatosensory System | Primary Somatosensory Cortex-L | Somatosensory System | Primary Somatosensory Cortex Trunk-L | 4.8050 |
| Olfactory System | Lateral Entorhinal Cortex Internal part-R | Limbic System | PreLimbic System-L | 4.7112 |
| Olfactory System | Glomerular Layer of the Accessory Olfactory Bulb-R | Parietal System | Parietal Cortex Postero Dorsal Part-L | 4.4016 |
| Olfactory System | Lateral Entorhinal Cortex external part-R | Hippocampus Fomation | Presubiculum-L | 4.5716 |
| Sensory-Motor | Molecular Layer of the Cerebellum-R | Parietal System | Parietal Cortex Postero Rostral-R | 4.5499 |
| Visual System | Medio Lateral Secondary Visual Cortex-L | Motor System | Primary Motor Cortex-L | 4.4743 |

## 2.6 Supplementary table S6. ROI-wise Functional Connectivity Between Groups

| **Seed Region** | **Connective Region** | **Voxels** | **Peak Intensity** | **Peak Coordinate** | | | **FDR** |
| --- | --- | --- | --- | --- | --- | --- | --- |
|  |  |  |  | **X** | **Y** | **Z** |  |
| **Sham vs Model (Decrese)** | | | | | | | |
| Frontal Association Cortex-L | PreLimbic System_R | 255 | 14.2736 | 5 | 24.95 | 18.2 | **Corrected** |
|  | Olfactory Bulb_L | 50 | 11.9425 | -1 | 45.95 | 9.2 |  |
| Frontal Association Cortex-R | PreLimbic System_R | 147 | 12.7616 | 5 | 24.95 | 18.2 |  |
|  | Primary Cingular Cortex_R | 32 | 7.4865 | 8 | 45.95 | 45.2 |  |
|  | Olfactory Bulb_L | 22 | 9.7127 | -40 | 27.95 | -8.8 |  |
|  | Cornu Ammonis 2_R | 17 | 8.6357 | 59 | -47.05 | 6.2 |  |
|  | Retrosplenial Dysgranular Cortex_R | 12 | 6.0438 | 2 | -14.05 | 60.2 |  |
| PreLimbic System-L | Primary Cingular Cortex-L | 115 | 9.5058 | -13 | 21.95 | 51.2 | **Corrected** |
|  | Olfactory Bulb-R | 10 | 5.9765 | 17 | 51.95 | 24.2 |  |
| PreLimbic System-R | Secondary PreLimbic System-R | 102 | 9.4843 | 8 | 27.95 | 30.2 |  |
|  | Olfactory Bulb-R | 30 | 9.0921 | 17 | 48.95 | 21.2 |  |
|  | Primary Cingular Cortex-L | 18 | 6.4730 | -16 | 18.95 | 48.2 |  |
| Olfactory Bulb-L | Primary Cingular Cortex-L | 142 | 12.8212 | -1 | 24.95 | 48.2 | **Corrected** |
|  | Retrosplenial Dysgranular Cortex-R | 38 | 10.6148 | 5 | -59.05 | 66.2 |  |
|  | PreLimbic System-R | 38 | 9.3160 | 2 | 39.95 | 12.2 |  |
| Olfactory Bulb-R | Primary Cingular Cortex-R | 20 | 11.7997 | -43 | 9.95 | -11.8 | **Corrected** |
|  | Entorhinal Cortex-L | 16 | 7.6861 | -22 | -12.95 | -2.8 | **Corrected** |
| Primary Cingular Cortex-L | PreLimbic System-R | 273 | 15.2230 | 5 | 27.95 | 24.2 | **Corrected** |
| Primary Cingular Cortex-R | Secondary Cingular Cortex-R | 276 | 12.0761 | 5 | 21.95 | 36.2 |  |
|  | PreLimbic System_R | 11 | 9.4491 | 38 | 33.95 | 3.2 |  |
|  | Primary Cingular Cortex-R | 11 | 16.5615 | 11 | 45.95 | 45.2 |  |
| Secondary Cingular Cortex-L | PreLimbic System-R | 167 | 8.5502 | 2 | 54.95 | 27.2 |  |
|  | Olfactory Bulb-L | 23 | 7.3026 | -28 | 36.95 | 6.2 |  |
|  | Primary Cingular Cortex-L | 17 | 6.9817 | -1 | 0.95 | 57.2 |  |
| Secondary Cingular Cortex-R | PreLimbic System-R | 188 | 12.1944 | 59 | -47.05 | 6.2 |  |
|  | Cornu Ammonis 2-R | 23 | 10.2630 | 2 | 48.95 | 33.2 |  |
| Cornu Ammonis 3-L | Entorhinal Cortex-L | 10 | 7.7182 | -52 | -32.95 | -26.8 |  |
| Cornu Ammonis 3-R | Parasubiculum-R | 16 | 5.7980 | 44 | -68.05 | 27.2 |  |
|  | Entorhinal Cortex-L | 15 | 6.6887 | -55 | -20.05 | -23.8 |  |
| Cornu Ammonis 2-R | Entorhinal Cortex-L | 13 | 13.2857 | -55 | -11.05 | -26.8 |  |
| Subiculum-L | Primary Somatosensory Cortex Barrel field-R | 11 | 8.0037 | 59 | -32.95 | 33.2 |  |
| Subiculum-R | Primary Cingular Cortex-R | 19 | 9.9689 | 11 | 48.95 | 48.2 |  |
|  | PreLimbic System-L | 11 | 7.4589 | -4 | 45.95 | 21.2 |  |
| Fasciola Cinereum-L | Retrosplenial Dysgranular Cortex-R | 12 | 6.4459 | 8 | -56.05 | 63.2 |  |
| Fasciola Cinereum-R | Corpus Callosum and Associated Subcortical White Matter-R | 17 | 7.6550 | 2 | -3.95 | 33.2 |  |
| Retrosplenial Dysgranular Cortex-L | Olfactory Bulb-L | 11 | 13.9886 | -22 | 42.95 | -5.8 | **Corrected** |
| Retrosplenial Dysgranular Cortex-R | PreLimbic System-R | 11 | 8.0472 | 11 | 33.95 | 21.2 |  |
|  | Olfactory Bulb-R | 10 | 6.0311 | 5 | 60.95 | 33.2 |  |
| Agranular Dysgranular Insular Cortex_L | PreLimbic System_L | 16 | 9.4047 | -7 | 24.95 | 18.2 |  |
| Agranular Dysgranular Insular Cortex _R | Primary Cingular Cortex_R | 56 | 10.3434 | 8 | 15.95 | 51.2 |  |
|  | Corpus Callosum and Associated Subcortical White Matter_L | 31 | 9.2590 | -34 | -62.05 | 48.2 |  |
|  | PreLimbic System_R | 20 | 8.9420 | 5 | 51.95 | 18.2 |  |
|  | Olfactory Bulb_L | 18 | 7.7021 | -16 | 54.95 | 21.2 |  |
| Bed Nucleus of the Stria Terminalis_L | Cornu Ammonis 1_R | 21 | 8.9517 | 53 | -56.05 | 33.2 |  |
| Bed Nucleus of the Stria Terminalis_-R | Striatum_R | 20 | 7.6880 | 20 | 15.95 | 6.2 |  |
| **Model vs CCYC (Increse)** | | | | | | | |
| Frontal Association Cortex-L | Cornu Ammonis 3_ R | 18 | -7.6029 | 41 | -53.05 | 30.2 |  |
|  | Subiculum_ L | 18 | -7.5376 | -7 | -47.05 | 39.2 |  |
|  | Retrosplenial Dysgranular Cortex_L | 11 | -6.4481 | -10 | -41.05 | 66.2 |  |
| Frontal Association Cortex-R | Retrosplenial Dysgranular Cortex_R | 14 | -5.7531 | 8 | -53.05 | 69.2 |  |
| PreLimbic System-R | Cornu Ammonis 1-L | 10 | -4.8157 | -61 | -56.05 | 9.2 |  |
|  | Corpus Callosum and Associated Subcortical White Matter-R | 11 | -5.7312 | 65 | 535.05 | 15.2 |  |
| Olfactory Bulb-L | Corpus Callosum and Associated Subcortical White Matter-R | 17 | -6.4970 | 65 | -47.05 | 12.2 |  |
| Olfactory Bulb-R | Molecular Layer of the Cerebellum-R | 15 | -6.2575 | 23 | -89.05 | -23.8 |  |
|  | Retrosplenial Granular Cortex Part B-R White Matter-R | 15 | -5.1559 | 5 | -20.05 | 45.2 |  |
| Primary Cingular Cortex-L | Subiculum-L | 13 | -6.7937 | -40 | -65.05 | 39.2 |  |
| Primary Cingular Cortex-R | Parasubiculum_L | 41 | -9.9077 | 29 | -65.05 | 60.2 |  |
|  | Corpus Callosum and Associated Subcortical White Matter-R | 16 | -7.1257 | 38 | -74.05 | 45.2 |  |
| Secondary Cingular Cortex-L | Cornu Ammonis 2-R | 10 | -6.3724 | 59 | -50.05 | 9.2 |  |
|  | Cornu Ammonis 3-R | 7 | -6.4692 | 47 | -47.05 | 21.2 |  |
| Secondary Cingular Cortex-R | Cornu Ammonis 2-R | 11 | -5.3632 | 62 | -47.05 | 9.2 |  |
| Cornu Ammonis 3-L | Olfactory Bulb-L | 5 | -5.9065 | -10 | 60.95 | 18.2 |  |
| Cornu Ammonis 3-R | Entorhinal Cortex-L | 16 | -5.9828 | -37 | 3.95 | -20.8 |  |
|  | Corpus Callosum and Associated Subcortical White Matter-R | 15 | -5.2927 | 14 | 21.95 | 30.2 |  |
| Cornu Ammonis 2-L | Olfactory Bulb-L | 5 | -6.9136 | -22 | 33.95 | 6.2 |  |
| Cornu Ammonis 2-R | Corpus Callosum and Associated Subcortical White Matter-R | 7 | -4.2350 | 8 | -44.05 | -17.8 |  |
| Subiculum-L | Corpus Callosum and Associated Subcortical White Matter-R | 13 | -7.3003 | 8 | -38.05 | 48.2 |  |
| Fasciola Cinereum-L | Retrosplenial Dysgranular Cortex-R | 10 | -5.9220 | 8 | -47.05 | 66.2 |  |
| Retrosplenial Dysgranular Cortex-L | Retrosplenial Dysgranular Cortex-R | 11 | -8.1197 | 14 | -62.05 | 63.2 |  |
| Agranular Dysgranular Insular Cortex_L | Corpus Callosum and Associated Subcortical White Matter-R | 16 | -7.1257 | 38 | -74.05 | 45.2 |  |
| Bed Nucleus of the Stria Terminalis_-R | Retrosplenial Dysgranular Cortex_R | 18 | -6.9408 | 20 | -68.05 | 51.2 |  |
|  | Parasubiculum_L | 11 | -12.2357 | -37 | -65.05 | 33.2 |  |
